# Hair-based biomarkers in women with major depressive disorder: Glucocorticoids, endocannabinoids, *N*-acylethanolamines, and testosterone

**DOI:** 10.1016/j.cpnec.2021.100068

**Published:** 2021-06-21

**Authors:** Alexander Behnke, Anja M. Gumpp, Aniko Krumbholz, Alexandra M. Bach, Gustav Schelling, Iris-Tatjana Kolassa, Roberto Rojas

**Affiliations:** a Clinical & Biological Psychology, Institute of Psychology and Education, Ulm University, DE-89081, Ulm, Germany; b Institute of Doping Analysis and Sports Biochemistry (IDAS) Dresden, DE-01731, Kreischa, Germany; c Department of Anaesthesiology, Ludwig Maximilians University, DE-82131, Munich, Germany; d University Psychotherapeutic Outpatient Clinic, Institute of Psychology and Education, Ulm University, DE-89073, Ulm, Germany

**Keywords:** Major depressive disorder, Hair cortisol, Cortisone, Testosterone, Endocannabinoids, Mass spectrometry

## Abstract

**Background:**

Stress-related alterations in the regulation of several endocrine systems, including the hypothalamus-pituitary-adrenal (HPA) and -gonadal (HPG) axes and the endocannabinoid system are proposed to be involved in the etiology of major depressive disorder (MDD). Therefore, this study examines whether altered concentrations of glucocorticoids, testosterone, endocannabinoids, and related *N*-acylethanolamines accumulated in hair are present in MDD.

**Methods:**

Female participants (range: 19–59, *Mdn* = 30.5 years) were recruited, including *n* = 21 with a current MDD episode and *n* = 27 nondepressed controls without any current mental disorder. Weight-standardized samples of 3 cm hair segments were analyzed which equals to three months of retrospectively assessed biomarkers in hair. Concentrations of cortisol, cortisone, testosterone, endocannabinoids (i.e., anandamide [AEA], 2-arachidonylglycerol [2-AG]), and selected *N*-acylethanolamines (i.e., stearoylethanolamide [SEA], oleoylethanolamide [OEA], palmitoylethanolamide [PEA]) were measured using mass spectrometry.

**Results:**

Female MDD patients exhibited lower cortisol and cortisone levels in hair than nondepressed controls, whereas the hair concentrations of endocannabinoids, *N*-acylethanolamines, and testosterone did not differ between the groups.

**Conclusions:**

Our results add to the heterogeneous body of findings on alterations in hair-stored glucocorticoids and endocannabinoids in MDD. As in previous studies, there was no evidence of altered testosterone concentrations in the hair of MDD patients. Larger and longitudinal studies are needed to comprehensively explore the overall picture of endocrine alterations in MDD.

## Highlights

• Analysis of hair-based markers of endocrine alterations in female MDD outpatients.
• Lower hair cortisol and cortisone concentrations in mild to moderately severe MDD.
• No differences in endocannabinoid, *N*-acylethanolamine, and testosterone hair levels.
• Testosterone and cortisone levels were linked to higher stress/trauma exposure.

## 1 Introduction

Stress-related alterations in the regulation of the body’s endocrine system such as the Hypothalamus-Pituitary-Adrenal (HPA) axis, the Hypothalamus-Pituitary-Gonadal (HPG) axis, and the endocannabinoid system are indicated to be involved in the etiology of MDD [[1], [2], [3]]. The HPA axis is a major regulator of the physiological stress reaction [4]. In humans upon stress exposure, the HPA axis triggers the adrenals to release glucocorticoids (mainly cortisol) to induce pleiotropic effects. Cortisol promotes the cellular energy production, primes the innate immune system to respond to injury and infection, and it exerts immunosuppressive and anti-inflammatory effects. In a negative feedback loop, increasing cortisol concentrations modulate the HPA-axis activity to inhibit the further release of glucocorticoids [4]. Recent reviews suggest that MDD involves a state of hypercortisolism (i.e., elevated cortisol circulation in blood, salvia, and urine) along with desensitized glucocorticoid receptors and reduced HPA-axis reactivity to psychosocial stress (HPA-axis alterations in MDD are extensively reviewed in e.g., [5,6]).

Circulating cortisol is believed to accumulate in hair over a period of weeks to months. Therefore, assuming that hair grows about 1 cm per month, the retrospective cortisol output over the previous three months can be quantified in the scalp-nearest 3 cm of hair [7,8]. Studies measuring hair cortisol concentrations (HCC) provided inconsistent evidence of higher [9,10], lower [11,12], and unchanged HCC [13,14] in patients with MDD when compared to nondepressed controls (for a meta-analysis see [15]). However, these studies examined heterogeneous cohorts which differed considerably in depression chronicity, sex, and age (for comprehensive overviews on HCC alterations in stress states and mental health beyond MDD see [16,17]). According to a meta-analysis [17], studies in nonclinical cohorts found no consistent associations between HCC and self-reported depressive symptom severity. Moreover, recent longitudinal studies did not confirm prospective associations between HCC alterations and changes in depressive symptoms [18,19]. Once secreted by the adrenals, cortisol can be enzymatically converted to its inactive form, cortisone. Compared to research on HCC, investigations of hair cortisone concentrations are rare. In a large cohort study, Gerritsen et al. [13] observed no difference in hair cortisone levels between MDD patients and nondepressed controls.

HPA-axis activation and downregulation are pivotally regulated by endocannabinoids [3,[20], [21], [22]], i.e., bioactive signaling molecules that are synthesized on demand from central nervous and peripheral cells [23,24]. Moreover, endocannabinoids are of importance in regulating immune activity [1,23]. Activated leukocytes secrete the endocannabinoids anandamide (AEA) and 2-arachidonylglycerol (2-AG) as well as chemically closely related *N*-acylethanolamines (NAEs) such as stearoylethanolamide (SEA), oleoylethanolamide (OEA), and palmitoylethanolamide (PEA) [24] to confine the activation of further immune cells [1,23]. Due to their importance in regulating both the endocrine stress response and the immune system (for comprehensive overviews on endocannabinoid and NAE alterations in stress states and mental health see e.g., [1,20]), endocannabinoids and NAEs could be highly relevant for the etiology of stress-related mental health problems such as MDD [3,22]. So far, three studies have investigated endocannabinoid and NAE hair concentrations in MDD. More intense depressive symptoms were associated with higher 2-AG hair concentrations in male adolescent refugees from Syria [25] and with higher SEA and OEA levels in hair of German rescue workers [26]. Conversely, in a German population sample, AEA, 2-AG, SEA, OEA, and PEA concentrations in hair did not correlate with the severity of subclinical depressive symptoms [27].

Furthermore, the HPA axis is involved in complex interaction with the HPG axis. In short, testosterone dampens the stress-related reactivity of the HPA axis along with peripheral stress correlates; vice versa, stress exposure inhibits the release of gonadotrophins, which results in the suppression of estrogen and testosterone production [2,28]. Due to its influence on the HPA axis, altered testosterone levels could be relevant in stress-related mental disorders such as MDD [2]. Preliminary clinical studies indicate the combined administration of estradiol and testosterone to female MDD patients alleviated their depressive and anxiety symptoms [[29], [30], [31]]. However, hair testosterone concentrations (HTC) showed negligible negative associations with depressive symptoms in a population-based sample of Indian women [32], and, in a clinical study with male and female participants, HTC did not differ between MDD patients and non-depressed controls [33]. Longitudinal studies could not corroborate associations between HTC alterations and the course of depressive symptoms among men or women at different ages [34,35].

Taken together, there is conflicting evidence on alterations in the regulation of glucocorticoids, testosterone, endocannabinoids, and NAEs in MDD. To date, no study has simultaneously examined all these endocrine markers in hair for possible alterations in MDD. Because of conflicting findings on alterations in glucocorticoid, endocannabinoid, and NAE levels in hair, this study explores corresponding differences between patients with clinical MDD and nondepressed controls. Previous studies observed no alterations in testosterone concentrations in the hair of MDD patients; therefore, we do not expect to find major differences related to MDD.

## 2 Methods

### 2.1 Participants and procedure

For this study, depressed women were recruited from the waiting list of the Ulm University Psychotherapeutic Outpatient Clinic (Ulm, Germany). Single additional depressed women and nondepressed controls were recruited via public advertisements in newspapers and town (see [36] for further details). Forty-eight women were eligible for study participation (age: 19–59 years, *Mdn* = 30.5, *IQR* = 22). All study participants received comprehensive information about the study procedures and declared their written informed consent. For participation, they received a remuneration of €40. All study procedures were in accordance with the Declaration of Helsinki and have been approved by the Ulm University ethics committee. Criteria for study inclusion were (i) female sex; (ii) age between 18 and 60 years; (iii) absence of neuropsychiatric and severe physical diseases (e.g., cancer, hepatitis, HIV); (iv) absence of immunomodulatory medication (e.g., systemic glucocorticoids). Trained clinical staff conducted standardized interviews with the participants to assess their (i) sociodemographic data and medical history; (ii) mental health condition; (iii) lifetime exposure to traumatic experiences. Clinical diagnoses were determined by licensed psychotherapists on the basis of the German Structured Clinical Interview for DSM-IV-TR (SCID [37]; updated according to DSM-5). Out of the 48 women eligible for participation, 21 of them fulfilled the criteria of a current MDD episode according to DSM-5 (MDD group; symptom severity: *n* = 3 high, *n* = 11 moderate, *n* = 7 mild), and 27 had no current mental disorder (nondepressed controls). According to the clinicians’ judgment, all MDD patients retained a relatively high psychosocial functioning level, thus meeting the eligibility to receive outpatient psychotherapy.

### 2.2 Hair sampling and analysis

Trained scientific staff collected samples by cutting three hair strands of at least 3 cm length from a posterior vertex position of the scalp. At the time of hair collection, hair-related data were assessed, including natural hair color, frequency of hair washing, and hair treatments. The groups did not differ with respect to these variables (Table 1). Following a standardized protocol [38], hair strands were cut to 3 cm segments, and a standardized amount (weight: 9.7–10.2 mg, *Med* = 10.0, *IQR* = 0.1) was stored in sterile Eppendorf tubes.

**Table 1:** Sample characteristics.

	Nondepressed controls (*n* = 27)	Major depressive disorder (*n* = 21)	
	*Mdn* (*IQR*) [Min, Max] /*n* (%)	*Mdn* (*IQR*) [Min, Max] /*n* (%)	Test statistics
Age [years]	28.0 (16.0) [20.0, 57.0]	33.0 (27.0) [19.0, 59.0]	*U* = 567.5, *p* = .050
Body Mass Index (BMI)	23.0 (5.6) [18.6, 30.7]	25.5 (5.4) [18.0, 34.6]	*U* = 571.5, *p* = .061
Depressive symptom severity (BDI-II)	2.0 (3.5) [0.0, 10.0]	23.0 (6.0) [9.0, 51.0]	*U* = 379.5, ***p* < .001**
Child maltreatment experiences (CTQ)	29.0 (12.5) [25.0, 52.0]	43.0 (17.0) [27.0, 58.0]	*U* = 509.0, ***p* = .002**
Potentially traumatic life events (PDS)	0 (2) [0, 10]	2 (2) [0, 6]	*U* = 535.5, ***p* = .007**
Posttraumatic distress (PDS)	2 (5.5) [0, 13]	9 (10.0) [0, 34]	*U* = 483.5, ***p* < .001**
**Mental and physical health**			
Antidepressant medication ^a^	0 (0%)	12 (57.1%)	χ^2^ = 17.64, ***p* < .001**
Previous depressive episodes	0 (0) [0, 1]	3 (3) [0, 11^b^]	*U* = 411.0, ***p* < .001**
Concurrent comorbid mental health diagnosis ^c^	0 (0%)	8 (38.1%)	χ^2^ = 9.75, ***p* < .001**
Lifetime mental health diagnosis	8^d^ (29.6%)	20^e^ (95.2%)	χ^2^ = 8.73, ***p* = .001**
Chronic physical illness ^f^	4 (14.8%)	4 (19.0%)	χ^2^ = 0.00, *p* = .715
Irregular or missing menstrual cycle	3 (11.1%)	7 (33.3%)	χ^2^ = 2.32, *p* = .081
**Hair-related variables**			
Sample weight [mg]	10.1 (0.1) [9.7, 10.2]	10.0 (0.1) [9.9, 10.2]	*U* = 697.0, *p* = .410
Natural hair color ^g^	*n*_red-blond_ = 12 (44.4%), *n*_brown-black_ = 15 (55.6%)	*n*_red-blond_ = 10 (50.0%), *n*_brown-black_ = 10 (50.0%)	χ^2^ = 0.01, *p* = .773
Weekly hair washings	3.5 (1.0) [1.0, 7.0]	3.0 (1.5) [1.5, 7.0]	*U* = 705.5, *p* = .351
Hair dying ^g^	5 (19.2%)	9 (42.9%)	χ^2^ = 2.07, *p* = .112
Hair coloring	3 (11.1%)	4 (19.0%)	χ^2^ = 0.13, *p* = .683

*Note*: Group differences were analyzed with Mann-Whitney *U*-tests. Distributions of count data were compared using Fisher's exact test. ^a^ Patients with antidepressant medication received Bupropion, Escitalopram, Mirtazapine, Trimipramine, Fluoxetine, Venlafaxine, Duloxetine, and combinations thereof, respectively (i.e., Paroxetine/Escitalopram, Trimipramine/Escitalopram, Mirtazapine/Escitalopram, Venlafaxine/Pregabalin, and Pregabalin/Sertraline/Bupropion/Trazodone). ^b^ Two participants could not remember the exact number of previous MDD episodes; however, the number was higher then 10. ^c^ Eight MDD patients were also diagnosed with specific phobia (*n* = 3), social phobia (*n* = 2), binge eating disorder, and/or agoraphobia with and without panic disorder. ^d^ Lifetime diagnoses of nondepressed controls include depressive episodes (*n* = 3), adjustment disorder (*n* = 2), agoraphobia with and without panic disorder, and spider phobia (each *n* = 1). ^e^ In the MDD group, all lifetime diagnoses involved MDD (*n* = 20), including cases with remitted anxiety disorders (*n* = 11, i.e., specific or social phobia, panic disorder with agoraphobia), posttraumatic stress disorder (*n* = 4), and/or bulimia nervosa (*n* = 2). ^f^ Including *n* = 6 hypothyroidism with compensatory medication. ^g^ One missing value.

Hair analysis was performed using a validated protocol with applying small adaptations [7]. Hair samples were powdered using a ball mill (Homogenizer FastPrep-24, MP Biomedicals, USA). The internal standard was added in every hair sample, calibration point, and blank sample. The internal standard contained cortisol-d_4_ (CIL, Andover, USA), cortisone-d_8_ (Sigma-Aldrich, Missouri, USA), nandrolone-d_3_ (NMI, Pymble, Australia), AEA-d_4_, PEA-d_4_, OEA-d_4_, SEA-d_3,_ and 2AG-d_5_ (last five: Cayman Chemical, Ann Arbor, USA). Extraction was carried out in the Eppendorf tubes with 1.5 mL methanol (MeOH, gradient grade, J.T.Baker, Deventer, Netherlands) in an ultrasonic bath at 50 °C for 6 h. After centrifugation (3100 rpm, 10 min, 20 °C) the liquid phase was separated and 30 μl ethylene glycol (J.T.Baker, Deventer, Netherlands) was added. The supernatant was dried under nitrogen stream at 60 °C. After reconstitution with 500 μl of water, a solid-phase extraction (SPE; Aspec XL4, Gilson, Middleton, USA) was applied on Bond Elut Plexa columns (3 mL, 60 mg; Agilent Technologies, Böblingen, Germany). The SPE procedure is detailed in Krumbholz et al. [7]. Hair residues from methanol extraction were hydrolyzed with 1.0 mL 0.5 N KOH-solution (potassium-hydroxide pellets, 85%–90%, VWR, Germany) for 18 h at 60 °C. After centrifugation (3100 rpm, 10 min, 20 °C), the liquid phase was separated and clean-up was performed by SPE again. Extracts from both SPE runs were combined. After adding 30 μl ethylene glycol, samples were evaporated and reconstituted with HPLC-buffer A. Hair concentrations were measured by HPLC-HR-MS/MS-technology using an Agilent HPLC system 1290 infinity and a Sciex TripleTOF 6600 mass spectrometer. Chromatographic separation was operated on an Agilent ZORBAX-Eclipse XDB-C8-column using a linear gradient using the HPLC-buffer A (water/acetonitrile (95:5), 2 mmol NH_4_AC, 0.1% acetic acid) and HPLC-buffer B (water/acetonitrile (5:95), 2 mmol NH_4_Ac, 0.1% acetic acid). The minimum detection limits of the described protocols were: 2 pg/mg for cortisol and cortisone; 3 pg/mg for testosterone; 4.3 pg/mg for progesterone; 1 pg/mg for AEA; 50 pg/mg for 2 AG, SEA, and OEA; 100 pg/mg for PEA. Intra- and interday precision were calculated according to international guidelines (GTFCh) and were ±10%. Raw data of measured biological hair parameters are presented in Table 1. AEA and progesterone concentrations could not be detected in a sufficient number of samples (*n* = 0 and 9, respectively) and were therefore neglected subsequently.

### 2.3 Clinical measures

On the German Beck’s Depression Inventory version II (BDI, [39]), participants rated the severity of their depressive symptoms within the two weeks prior to participation. Moreover, they reported their exposure to child maltreatment on the German Childhood Trauma Questionnaire (CTQ, [40]), and their lifetime exposure to potentially traumatic events as well as their current posttraumatic stress symptoms on the German Posttraumatic Diagnostic Scale (PDS, [41]). MDD patients exhibited higher values than nondepressed controls on the abovementioned inventories, had a higher number of comorbid concurrent and lifetime mental health diagnoses, and 57% of them took antidepressant medication (Table 1).

### 2.4 Statistical analyses

Statistical analyses were conducted with R 3.6.2 [42]. Nonparametric and robust statistical methods were applied, as the majority of variables was nonnormally distributed and/or exhibited influential data points. Groups were compared using Mann-Whitney *U*-tests and variable associations were analyzed with Spearman correlations (*r*_S_). Group comparisons were adjusted for age, BMI, and the frequency of hair washing using 10,000-fold bootstrapped robust MM-estimator-based linear models [43] (using the *robustbase* package, [44]) if Spearman correlations indicated significant associations between any of these covariates and the hair parameters. MM-estimator based ANCOVAs are characterized by a high breaking point with respect to non-normally distributed data and remote data points [43]. The false discovery rate (FDR) has been used to adjust *p*-values for multiple comparisons. Partial Spearman correlations (*r*_S_’) were computed to adjust variable associations for relevant influences of age, BMI, and/or the frequency of hair washing (using the *ppcor* package, [45]). Power calculation was performed for a Mann-Whitney *U* test, and assuming α < 0.05 and 1-β = 0.80, a minimal effect size of *d* = 0.67 can be detected at the given sample size.

## 3 Results

### 3.1 Glucocorticoids

**Fig. 1:**
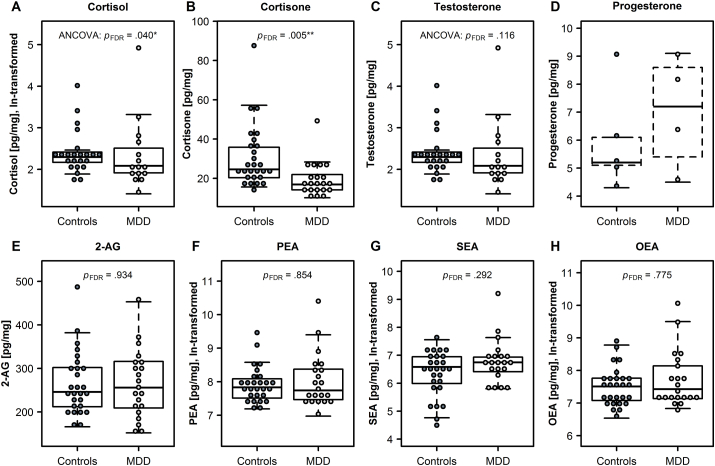
Results of Mann–Whitney *U*-tests and robust ANCOVAs on differences in glucocorticoids, sex hormones, endocannabinoids, and *N*-acylethanolamines between female major depressive disorder (MDD) outpatients and nondepressed controls. ANCOVAs were computed to consider the influences of age and/or hair washing frequency as covariates. Progesterone concentrations were not statistically analyzed due to the small number of successful measures.

As correlation analyses indicated that HCC were lower among women who more frequently washed their hair (*r*_S_ = −0.48, *p* = .002), subsequent analyses of HCC were adjusted for this variable. A robust ANCOVA revealed MDD patients exhibited significantly lower HCC than nondepressed controls (Table 2, Fig. 1A) and HCC were negatively correlated with depressive symptom severity as a trend (*r*_S_’ = −0.33, *p* = .047). Moreover, MDD patients exhibited lower hair cortisone levels than nondepressed controls (Table 2; Fig. 1B). Hair cortisone levels were also lower among women who reported higher depressive (*r*_S_ = −0.39, *p* = .006) and posttraumatic stress symptoms (*r*_S_ = −0.30, *p* = .040) as well as more child maltreatment exposure (*r*_S_ = −0.33, *p* = .020).

**Table 2:** Group comparisons of hair variables.

	Nondepressed controls (*n* = 27)	Major depressive disorder (*n* = 21)		
	*Mdn* (*IQR*) [Range]	*Mdn* (*IQR*) [Range]	Test statistics	Effect size
Cortisol [pg/mg]^a^	9.7 (3.2) [4.6, 65.9]	8.3 (6.3) [3.9, 157.0]	ANCOVA^c^: *b*[95% CI]^d^ = −0.20 [ −0.36, −0.03], ***p* = .012, *p***_**FDR**_**= .040**	*d* = −.49
Cortisone [pg/mg]	24.6 (15.6) [15.6, 87.8]	16.9 (7.8) [10.1, 50.4]	*U* = 824.5, ***p* < .001**, ***p***_**FDR**_**= .005**	*r*_rank__biserial_ = −.57
Testosterone [pg/mg]	6.0 (3.8) [3.1, 23.1]	6.9 (2.4) [4.9, 12.5]	ANCOVA^c^: *b*[95% CI] = −0.95 [ −0.18, 2.13], *p* = .050, *p*_FDR_ = .116	*d* = .42
Progesterone [pg/mg]^b^	5.2 (1.0) [4.3, 9.1]	7.2 (2.5) [4.5, 9.1]	–	–
2-AG [pg/mg]	246 (90) [166, 483]	256 (107) [152, 453]	*U* = 657.5, *p* = .934, *p*_FDR_ = .934	*r*_rank__biserial_ = .01
PEA [pg/mg]	2522 (1450) [1323, 11815]	2302 (2586) [1069, 31671]	*U* = 645.0, *p* = .732, *p*_FDR_ = .854	*r*_rank__biserial_ = .06
SEA [pg/mg]	723 (634) [84, 1912]	851 (416) [331, 10007]	*U* = 595.0, *p* = .167, *p*_FDR_ = .292	*r*_rank__biserial_ = .23
OEA [pg/mg]	1829 (1168) [690, 7167]	1687 (2175) [927, 25327]	*U* = 633.0, *p* = .554, *p*_FDR_ = .775	*r*_rank__biserial_ = −.10

*Note*: Substance concentrations could not be measured in all hair samples: ^a^*n* = 23 vs. 15, ^b^*n* = 5 vs. 4; due to the small number of successful measures, progesterone concentrations were not statistically analyzed. ^c^ Robust ANCOVAs were adjusted for age and/or hair washing frequency. Detailed model and predictor statistics are provided in Supplementary Table 2^d^ The outcome variable’s natural logarithm (ln) was used to lower the influence of distant data points.

### 3.2 Endocannabinoids and NAEs

Mann-Whitney *U*-tests indicated the hair concentrations of 2-AG, PEA, SEA, and OEA did not differ between the groups (Table 2; Fig. 1). Moreover, these hair variables neither correlated with depressive symptom severity nor with any of the other psychological variables assessed (Supplementary Table 1).

### 3.3 Testosterone

Higher HTC correlated with higher age (*r*_S_ = 0.37, *p* = .010) and less hair washes (*r*_S_ = −0.37, *p* = .009). A robust ANCOVA adjusting for age and hair washing indicated MDD patients and nondepressed controls did not differ in their HTC (Table 2, Fig. 1C). However, partial correlations indicated HTC were higher in women who reported more subclinical posttraumatic stress symptoms (*r*_S_’ = 0.35, *p* < .001) as well as in those who were exposed to more traumatic events (*r*_S_’ = 0.21, *p* = .041) and child maltreatment (*r*_S_’ = 0.39, *p* < .001; see also Supplementary Table 1).

### 3.4 Inter-correlation of hair-based biomarkers

The concentrations of cortisol and cortisone correlated positively (*r*_S_’ = 0.61, *p* < .001), whereas the levels of cortisol and 2-AG (*r*_S_’ = −0.35, *p* = .031) as well as of cortisone and testosterone (*r*_S_’ = −0.25, *p* = .017) were negatively correlated. Moreover, PEA, SEA, and OEA levels were positively interrelated (*r*_S_ = 0.34–0.87, *p*’s ≤ 0.018; see also Supplementary Table 1).

## 4 Discussion

This study investigates the concentrations of HPA- and HPG-axis hormones, endocannabinoids, and selected NAEs in 3 cm-hair samples to provide a multi-biomarker perspective on endocrine alterations in female MDD outpatients. Women with a MDD episode showed lower levels of cortisol and cortisone than nondepressed controls. In line with this, previous studies reported lowered cortisol concentrations in hair of MDD patients [11,12], although existing findings on HCC in MDD are inconsistent [15]; i.e., studies also observed increased [9,10] and unchanged HCC among MDD patients [13,14]. Furthermore, studies in nonclinical cohorts reported no consistent cross-sectional and longitudinal associations between HCC and depressiveness [[17], [18], [19]]. However, corroborating the view of lowered glucocorticoid concentrations in MDD, in our study, women with MDD also showed lower hair concentrations of the metabolically inactive glucocorticoid cortisone when compared to nondepressed controls. Conversely, Gerritsen et al. [13] found no difference in hair cortisone levels between depressed and healthy subjects.

Lower cortisol and cortisone concentrations as observed in this study could reflect a reduced adrenal secretion of glucocorticoids as well as a lowered activity of the HPA axis. Cortisol confines ongoing inflammatory activity by various mechanisms, i.e., by inhibiting the gene expression of pro-inflammatory molecules, by inducing apoptosis of immune cells, and by indirectly activating anti-inflammatory molecules [4]. Thus, persistently lowered glucocorticoid signals could possibly contribute to maintain low-grade chronic inflammatory activity [5] which has been observed in MDD patients as well as individuals with chronic stress and a history of child maltreatment [46,47]. Accordingly, in our study cohort, more severe child maltreatment experiences correlated with lower cortisone levels in hair. Indeed, exposure to stress and trauma can cause alterations in the regulation of the glucocorticoid system [48], although empirical evidence on how these alterations manifest in hair is heterogeneous [49]. One reason for inconsistency could be genetic polymorphisms that have been reported to modulate how stress and trauma exposure alters glucocorticoid levels in hair [38].

Beside genetic differences, the HPA axis is modulated by other endocrine mechanisms which we also examined in this study, including the endocannabinoid system [20]. However, MDD patients and nondepressed controls did not differ in their hair concentrations of endocannabinoids and selected NAEs. Previous studies using hair provided initial evidence for positive associations of subclinical depressiveness with higher 2-AG, SEA, and OEA concentrations [25,26], whereas another study found no correlation of depressive and anxiety symptoms with AEA, 2-AG, SEA, OEA, and PEA concentrations [27]. In contrast to our findings, previous studies reported reduced OEA, SEA, and PEA levels in hair in association with a higher lifetime trauma exposure among war-traumatized PTSD patients [50] as well as higher 1-AG levels and lower SEA levels in hair among postpartum women with a history of childhood maltreatment experiences compared to women without such experiences [51]. Furthermore, the latter study reported lower endocannabinoid and NAE concentrations among individuals with psychiatric lifetime diagnosis [51].

Endocannabinoids, especially 2-AG and AEA, are involved in the negative feedback mechanism of the HPA axis [3,20,21]. Thus, elevated endocannabinoid concentrations in MDD patients could contribute to stronger inhibition of HPA-axis activity and, hence, lowered glucocorticoid concentration in MDD. Indeed, we found a negative correlation between the cortisol and 2-AG concentrations in hair. On par with this, previous studies also observed negative associations between glucocorticoid and endocannabinoid concentrations in hair [7,26]. Furthermore, endocannabinoids and NAEs are involved in the regulation of inflammatory reactions [1,23]. Chronic low-grade inflammation in MDD [46,47] could possibly be held up by an altered co-regulation of glucocorticoids, endocannabinoids, and NAEs. However, as we could not measure inflammatory markers in hair samples, this study could not address the possible implications of steroids, endocannabinoids, and NAEs for the immune regulation in MDD. Thus, future research is required to characterize the dynamics of glucocorticoids, endocannabinoids, NAEs, and inflammatory markers, as well as their concordance with the course of MDD.

In this study, the testosterone concentration in hair neither differed between MDD patients and nondepressed controls nor did it correlate with depressive symptom severity. This finding aligns with previous studies that observed no difference between the HTC of MDD patients and healthy controls [33] as well as null correlations between HTC and subclinical depressiveness in larger community samples [32,34,35]. In sum, this and previous studies suggest that testosterone in *hair* presents no informative biomarker of depressive symptoms among healthy and clinical cohorts.

Testosterone and estrogens inhibit the HPA axis’ response to acute and recurrent stress exposure [2,28]. Thus, the proposed beneficial effects of sex hormone administration on mood as reported by preliminary clinical studies [[29], [30], [31]] could possibly originate from an inhibitory interplay of HPA and HPG axes. In line with this, we observed a negative correlation between the concentrations of testosterone and cortisone in hair. Moreover, in rodents, repeated and chronic stress inhibits the secretion of testosterone and estrogens [28]. Conversely, among our participants, higher HTC was linked to more traumatic experiences during childhood and in later life. Besides, in our cohort of mainly premenopausal women, we observed HTC to positively correlate with age, although the production of testosterone is reported to remain relatively stable after adolescence [52]. Since previous studies on HTC did not report possible associations with age, our finding indicates the need for additional research. To derive an in-depth understanding of the relevance of testosterone in MDD, future studies need to repeatedly examine testosterone levels in different biological specimens (i.e., hair, blood, saliva) of patients at different ages and varying exposure to stress and trauma.

In general, additional validation is necessary to ascertain the associations between hair concentrations of HPA- and HPG-axis hormones, endocannabinoids, and NAEs with those in blood, saliva, and urine. Although it is known that for example endocannabinoids can cross the blood brain barrier [53], no direct correlation has been found between ECs in plasma and in cerebrospinal fluid [54]. Plasma (spot-measurement) and hair samples (long-term marker) further cover different time windows and can provide complementary data on acute vs. chronic stress. To date, interpreting hair-based biomarkers remains challenging, as the physiological mechanisms of steroid and endocannabinoid/NAE incorporation into hair have not yet been sufficiently understood [7,8,16]. Therefore, future research needs to better characterize the deposition and abundance of lipids and steroids in hair as well as to evaluate their concordance with measures in other peripheral specimens.

### 4.1 Limitations

This study is limited by its cross-sectional correlative design. To avoid possible biases due to differences in the amount of hair used for analysis, hair samples were prepared with a standardized weigh of 10 mg. Biomolecule concentrations were assessed using mass spectrometry, as it allows a more sensitive assessment than immunoassays [55]. Nonetheless, AEA and progesterone could not be determined in a sufficient number of samples, as their concentrations were apparently below the detection limit. Barely detectable progesterone concentrations in hair could result from menopause and the use of hormonal contraceptives which greatly decrease blood levels of progesterone and could hence reduce its incorporation into hair. Thus, future studies need to comprehensively assess women’s reproductive hormonal state (see [56] for methodological recommendations). Furthermore, it cannot be ruled out that the antidepressant medication of several patients might have affected the endocrine markers investigated in this study [5]. Additional analyses showed that in this study, MDD patients showed no differences in hair biomarker concentrations depending on their antidepressant medication. Another limitation of this study is its small sample size, which is nevertheless within the range of previous studies in clinical settings. Due to the study’s statistical power, possible influencing factors such as lifetime trauma exposure and perceived stress could not be considered as covariates. Future studies are required to replicate and generalize our findings among male MDD patients as well as among patients of different MDD severity, chronicity, medication, and suicidality.

## 5 Conclusions

Female middle-aged MDD patients with a mainly moderately severe disorder episode exhibited lower glucocorticoid levels in hair than nondepressed controls, whereas endocannabinoid, NAE, and testosterone concentrations in hair were not altered in MDD. The results add to the heterogeneous body of evidence on the role of glucocorticoids, endocannabinoids, NAEs, and testosterone in MDD. Additional studies in larger samples with male and female patients and a wider range of symptom severity are necessary to advance the multi-biomarker perspective on MDD.

## Declaration of competing interest

The authors declare that they have no known competing financial interests or personal relationships that could have appeared to influence the work reported in this paper.
