# Supplementary material for: Hair-based biomarkers in women with major depressive disorder: Glucocorticoids, endocannabinoids, N-acylethanolamines, and testosterone
Source: Compr Psychoneuroendocrinol. 2021 Jun 21;7:100068. doi: 10.1016/j.cpnec.2021.100068 (PMC9216461; doi:10.1016/j.cpnec.2021.100068)
Supplement: Multimedia component 1 [file mmc1.docx]

Hair-based biomarkers in women with major depressive disorder: Glucocorticoids, endocannabinoids, *N*-acylethanolamines, and testosterone

— Supplementary Material —

Alexander Behnke*^a^, Anja M. Gumpp^a^, Aniko Krumbholz^b^, Alexandra M. Bach^a^, Gustav Schelling^c^, Iris-Tatjana Kolassa^a^, Roberto Rojas^d^

^a^ Clinical & Biological Psychology, Institute of Psychology and Education, Ulm University, DE-89081 Ulm, Germany.
^b^ Institute of Doping Analysis and Sports Biochemistry (IDAS) Dresden, DE-01731 Kreischa, Germany.
^c^ Department of Anaesthesiology, Ludwig Maximilians University, DE-82131 Munich, Germany.

^d^ University Psychotherapeutic Outpatient Clinic, Institute of Psychology and Education, Ulm University, DE-89073 Ulm, Germany.

* Corresponding author: Alexander Behnke, Clinical & Biological Psychology, Institute of Psychology and Education, Ulm University, Albert-Einstein-Allee 47, 89081 Ulm, Germany, Email: alexander.behnke@uni-ulm.de, Phone: +49 731 5026595.

***Supplementary Table 1***.
Results of correlation analyses

|  | Cortisol | Cortisone | Testosterone | 2-AG | PEA | SEA | OEA |
| --- | --- | --- | --- | --- | --- | --- | --- |
| Cortisol | — |  |  |  |  |  |  |
| Cortisone | .54^***^ (.61^***^) | — |  |  |  |  |  |
| Testosterone | .01 (.00) | -.24 (-.25^*^) | — |  |  |  |  |
| 2-AG | -.22 (-.35^*^) | .01 | .03 (.03) | — |  |  |  |
| PEA | .11 (.08) | .07 | -.08 (-.08) | -.06 | — |  |  |
| SEA | .14 (.11) | -.21 | -.20 (-.20) | -.20 | .34^*^ | — |  |
| OEA | .02 (-.08) | -.13 | .06 (.06) | -.00 | .87^***^ | .34^*^ | — |
| BDI-II sum score | -.23 (-.33^*^) | -.39^**^ | .16 (.16) | .06 | .04 | .10 | .04 |
| CTQ sum score | -.14 (-.28) | -.33^*^ | .39^**^ (.39^***^) | .19 | .15 | .14 | .25 |
| PDS trauma load | .09 (.04) | -.13 | .21 (.21^*^) | -.09 | -.03 | -.14 | -.01 |
| PDS sum score | -.03 (-.09) | -.30^*^ | .35^*^ (.35^***^) | .24 | -.02 | .06 | .01 |
| Age | .20 (.07) | -.10 | .37^*^ | -.01 | .03 | -.01 | -.01 |
| BMI | .11 (-.01) | -.13 | .20 (.16) | .07 | .08 | .06 | .05 |
| Hair washing | -.48^**^ | -.01 | -.37^**^ | -.23 | .03 | -.02 | -.06 |

*Note*: ^*^ *p* < .050, ^**^ *p* < .010, ^***^ *p* < .001, two-tailed, *N* = 38−48. Values in brackets indicate partial Spearman rank correlations adjusted (i) for frequent hair washing in case of cortisol and (ii) for age and frequent hair washing in case of testosterone.

***Supplementary Table 2***.
Results of robust MM-estimator-based 10,000 times bootstrapped ANCOVAs

| Outcome | Predictors | *b* | *SE*_boot_ | 95%CI_boot_(*b*) | *p*_Boot_ |
| --- | --- | --- | --- | --- | --- |
| Cortisol ^a^ | Intercept | 2.61 | 0.12 | [2.35, 2.81] | <.001 |
|  | Group | -0.20 | 0.08 | [-0.36, -0.03] | .012 |
|  | Hair washing | -0.10 | 0.03 | [-0.16, -0.04] | .002 |
|  | *Overall model statistics*: *F*(2, 35) = 7.94, *p* = .00143, R² = .203, *N* = 38 | | | | |
| Testosterone | Intercept | 6.23 | 1.15 | [3.80, 8.31] | <.001 |
|  | Group | 0.95 | 0.59 | [-0.18, 2.13] | .050 |
|  | Hair washing | -0.33 | 0.18 | [-0.66, 0.04] | .035 |
|  | Age [years] | 0.04 | 0.02 | [-0.01, 0.08] | .054 |
|  | *Overall model statistics*: *F*(3, 44) = 3.05, *p* = .038, R² = .185, *N* = 48 | | | | |

*Note*: ^a^ The outcome variable’s natural logarithm was used to lower the influence of distant data points.
